# Growth Performance, Carcass Traits and Meat Quality in Rabbits Fed with Two Different Percentages of Extruded Linseed

**DOI:** 10.3390/foods14101778

**Published:** 2025-05-16

**Authors:** Imen Daboussi, Nour Elhouda Fehri, Michela Contò, Marta Castrica, Safa Bejaoui, Alda Quattrone, Mohamed Amine Ferchichi, Marouen Amraoui, Souha Tibaoui, Giulio Curone, Daniele Vigo, Laura Menchetti, Alessandro Dal Bosco, Egon Andoni, Gabriele Brecchia, Sebastiana Failla, Bayrem Jemmali

**Affiliations:** 1 Institut National Agronomique de Tunis, Université de Carthage, Tunisie, 43 Av. Charles Nicolle, Tunis 1082, Tunisia; imen.daboussi@fao.org; 2 Department of Veterinary Medicine and Animal Sciences, University of Milan, Via dell’Università 6, 26900 Lodi, Italy; nour.fehri@unimi.it (N.E.F.); alda.quattrone@unimi.it (A.Q.); giulio.curone@unimi.it (G.C.); daniele.vigo@unimi.it (D.V.); gabriele.brecchia@unimi.it (G.B.); 3 Consiglio per la Ricerca in Agricoltura e l’Analisi Dell’Economia Agraria (CREA), Research Centre for Animal Production and Aquaculture, Via Salaria 31, 00015 Rome, Italy; michela.conto@crea.gov.it (M.C.); sebastiana.failla@crea.gov.it (S.F.); 4 Comparative Biomedicine and Food Science, University of Padova, Agripolis, Viale dell’Univesità 16, 35020 Legnaro, Italy; 5 Laboratory of Integrated Improvement and Development of Animal Productivity and Food Resources LR13AGR02, Mateur Higher School of Agriculture, University of Carthage, Mateur 7030, Tunisia; bejaouisafaa1990@gmail.com (S.B.); el.ferchichi.87@gmail.com (M.A.F.); amraouimarouene@gmail.com (M.A.); souhatibaoui@gmail.com (S.T.); jemmali.bayrem@gmail.com (B.J.); 6 School of Biosciences and Veterinary Medicine, University of Camerino, Via Circonvallazione 93/95, 62024 Matelica, Italy; laura.menchetti@unicam.it; 7 Department of Agricultural, Environmental and Food Science, University of Perugia, Borgo XX Giugno 74, 06124 Perugia, Italy; alessandro.dalbosco@unipg.it; 8 Faculty of Veterinary Medicine, Agricultural University of Tirana, Kodër Kamëz, 1029 Tirana, Albania; eandoni@ubt.edu.al

**Keywords:** New Zealand white rabbit, extruded linseed, growth performance, carcass traits, meat quality, fatty acid profile, lipid oxidation, n-3 polyunsaturated fatty acids, sensory attributes

## Abstract

This study evaluated the effect of two levels of extruded linseed (EL) in the diet on growth performance, carcass yield, and meat quality of growing rabbits. Sixty-nine New Zealand White male rabbits (*Oryctolagus cuniculus*) were assigned after weaning to three dietary groups: control (C), 2.5% EL (L2.5%), and 5% EL (L5%). At the end of the fattening period (from 37 to 93 days of age), rabbits were slaughtered. EL supplementation significantly reduced average daily weight gain (ADG) in the L5% group (*p* < 0.05), while other performance parameters were not significantly affected. Meat from the L5% group exhibited a higher fat content (*p* < 0.001) and lower water-holding capacity (*p* < 0.05) compared to the others. The fatty acid profile showed a significant increase in n-3 polyunsaturated fatty acids (PUFAs) and a decrease in n-6 PUFA (*p* < 0.05), resulting in a markedly reduced n-6/n-3 ratio (*p* < 0.001) in supplemented groups. EL supplementation also enhanced long-chain n-3 PUFA levels, particularly docosapentaenoic acid (DPA). Although lipid oxidation was slightly increased (*p* < 0.05), sensory attributes remained unaffected. These findings support EL supplementation as a nutritional strategy to increase the n-3 fatty acids in rabbit meat without compromising physical and sensory quality.

## 1. Introduction

The domestic rabbit (*Oryctolagus cuniculus*) is an important livestock species in meat production systems, with approximately 682,000 t produced in 2023, according to FAOSTAT data [1]. Asia remains the leading producing region, accounting for 61% of global output, primarily driven by China and North Korea. Europe is the second-largest producer, contributing around 20% of global production, with Spain, Italy, and France among the main European producers [1,2]. However, global rabbit meat production has experienced a steady decline in recent years, with a decrease of about 22% recorded in 2023 compared to 2021. Over the past three years, production declined in both Asia and Europe (14.4% and 9.8%, respectively), although this reduction was less pronounced than during the previous three-year period. In contrast, Africa accounts for approximately 16% of global production, 72% of which is concentrated in North Africa, where a modest increase of 12% was observed over the same period. Despite the overall downturn, rabbit farming continues to play an important economic role in various regions. In 2021, the global rabbit meat market was valued at USD 1.5 billion, with exports totalling 29,900 t and a value of USD 136.7 million. Spain led global exports, followed by China and France, while the primary importers were some European countries, particularly Belgium and Germany [2].

To counteract the ongoing decline in rabbit meat production and commercial appeal, it is essential to both reduce production costs, which remain relatively high compared to other livestock sectors, and enhance consumer acceptance [2,3,4].

Improving the efficiency of rabbit production is, therefore, essential to ensure profitability. Growth performance in rabbits directly impacts production efficiency, and diet plays a key role in modulating both growth and product quality. Optimizing feed composition is crucial to ensure adequate nutrient intake, support physiological development, and control production costs, especially under the pressure of rising feed prices [5]. Moreover, consumer demand is increasingly shifting toward healthier meat with improved nutritional and functional value. Rabbit meat is naturally lean, low in cholesterol, and rich in high-quality protein [6]. It also offers a favourable balance of saturated fatty acids (SFA), monounsaturated fatty acids (MUFA), and polyunsaturated fatty acids (PUFAs), including the essential fatty acids linoleic acid (LA, 18:2n-6) and alpha-linolenic acid (ALA, 18:3n-3) [7,8]. Due to these characteristics, rabbit meat is often considered a functional food [8]. In this context, enriching rabbit diets with nutritionally valuable compounds further enhances the health value of the meat by facilitating the incorporation of bioactive molecules. One of the most explored strategies over the past two decades has been the dietary fortification with n-3 PUFAs [9], particularly ALA, which has been shown to be efficiently incorporated into rabbit meat [10,11].

Several studies on dietary n-3 supplementation in rabbits have been summarised in recent reviews [9,12] and in a comprehensive comparison of plant-based sources, as reported by Peiretti [13]. In this context, linseed has been extensively studied as a dietary source of alpha-linolenic acid (ALA), provided in the form of whole seeds, oil, or processed forms [11,14,15,16,17,18].

However, for linseed inclusion in diets, the physical form of the seeds appears to be a crucial factor. Whole flaxseeds contain several anti-nutritional compounds, such as cyanogenic glycosides [14,18,19], phytic acid, mucilage, and trypsin inhibitors [14,20], which can inhibit pancreatic enzyme activity and limit the digestibility and absorption of valuable nutrients. Many of these anti-nutritional factors can be degraded through heat treatments, although cyanogenic glycosides are only partially reduced [19]. Thermo-mechanical extrusion appears to be the most effective method for limiting the presence of cyanogenic glycosides. Nevertheless, careful modulation of the extrusion process is required to preserve the integrity of the n-3 fatty acids contained in linseed, while achieving high digestibility and a reduction of more than 90% of anti-nutritional compounds [14,18,19].

The importance of increasing n-3 intake in human nutrition is increasingly supported by scientific evidence, as n-3 PUFAs serve as precursors for bioactive signalling molecules, such as prostaglandins, which are involved in various physiological functions. These fatty acids are particularly known for their anti-inflammatory properties and for their role in preventing cardiovascular conditions, including hypercholesterolemia-related heart attacks and strokes [21,22]. However, conversion of ALA into its long-chain derivatives, eicosapentaenoic acid (EPA, 20:5n-3) and docosahexaenoic acid (DHA, 22:6n-3), is limited in rabbits due to the low efficiency of endogenous elongation and desaturation processes [8,9,23]. This raises concerns regarding the actual nutraceutical value of omega-3-enriched rabbit meat, as ALA alone may not offer the same health benefits as its long-chain derivatives [24]. The primary nutritional advantage of animal-based foods, including meats from fish, poultry, and mammals, lies in their ability to provide preformed long-chain omega-3 fatty acids, which are not readily available from plant-based sources, except for certain algae [25]. Historically, research on long-chain omega-3 fatty acids has primarily focused on EPA and DHA, which are abundant in fish. However, recent studies have underscored the significance of docosapentaenoic fatty acid (DPA, 22:5 n-3), an intermediate long-chain fatty acid known for its potential health benefits, including cardiovascular protection and anti-inflammatory properties [21,26,27]. Mammalian animals have shown a greater accumulation of DPA compared to EPA and DHA [21,27], highlighting the relevance of investigating long-chain fatty acid accumulation in rabbit tissues.

The aim of this study was to evaluate the accumulation of n-3 PUFA in rabbit meat following dietary supplementation with extruded linseed, with particular emphasis on the incorporation of long-chain n-3 PUFAs, while also considering potential effects on productive performance and key physicochemical and organoleptic traits of the meat.

## 2. Materials and Methods

### 2.1. Animals and Diets

This study was carried out as part of the PRIMA project: “Omega Rabbit: food for health BenefIT”, funded by the European Union. The experimental trial took place at the farm of the High School of Agriculture of Mateur located in the north of Tunisia, where the farm’s veterinary supervisor monitored the rabbits’ health and welfare daily.

All efforts were made to minimise animal suffering and to use only the number of animals necessary to produce consistent results. After weaning, 69 New Zealand White male rabbits, all obtained from the same certified breeder, were individually housed in standard cages (39 cm in length, 37 cm in width, and 29 cm in height) under controlled environmental conditions, with temperatures ranging between 18 °C and 23 °C. The number of rabbits utilised in the study corresponded to the total number of male offspring weaned from 20 female breeding does, all of which were raised under uniform management conditions on the same farm.

At 37 days of age, the rabbits were randomly assigned to three dietary treatment groups (23 rabbits per group), each receiving a different diet pelleted along with fresh water.

The diets were formulated according to nutritional guidelines for fattening rabbits [28], with particular attention given to maintaining an appropriate balance between fibre and protein to metabolisable energy. Adequate fibre intake was ensured through the inclusion of alfalfa hay. All ingredients were thoroughly mixed and pelleted by a commercial feed manufacturer. The pellets had a standard diameter of 4 mm and a length of approximately 10–12 mm. After pelleting, the feed was dried, cooled, and stored under hygienic conditions to preserve quality and nutritional stability, ensuring a consistent composition of crude protein and digestible energy across all experimental groups. In the experimental diets, the additional energy and protein supplied by the extruded linseed were primarily offset by a proportional reduction in barley content.

The dietary treatments included the following: (i) a control diet consisting of commercial feed (C); (ii) the same commercial feed as the control group, supplemented with 2.5% EL (L2.5%); and (iii) the same commercial feed as the control group, supplemented with 5% EL (L5%). The formulation of the pelleted diets, along with their chemical composition and fatty acid profiles, is detailed in Table 1. The two levels of EL inclusion in the diet were selected based on recommendations reported in the literature [8,15,29].

Throughout the experimental period (from 37 to 93 days of age), the rabbits were fed a gradually increasing daily ration, starting at 100 g/day and reaching 160 g/day.

### 2.2. Growth Performance, and Slaughter Procedures and Meat Samples

The rabbits’ live weight (LW) and feed intake (FI) were recorded weekly to determine the average daily weight gain (ADG) by comparing the final weight to the initial weight. Once a week, all the rabbits were weighed with a rabbit scale with a basket (Spring Dial Rabbit Scale with Basket, Santee, CA, USA). Additionally, the feed conversion ratio (FCR) was assessed by evaluating the amount of feed consumed in relation to the weight gained.

The rabbits were slaughtered at approximately 93 days of age in an official slaughterhouse, following halal slaughtering procedures. In compliance with Law No. 2005-95 of 18 October 2005, regulating the organisation of animal husbandry and animal products, and its implementing regulations, the animals were slaughtered by trained personnel through severing the carotid artery, jugular vein, trachea, and oesophagus, without prior stunning [31,32]. Slaughter weight (SW) was recorded using a calibrated technical scale with precision to two decimal places.

Fifteen carcasses per group were randomly selected for further analysis. The number of animals per group used for evaluating slaughter performance and meat analysis was based on statistical determination, including power analysis, desired significance (0.80, 0.05, respectively) and the expected variability of the main tested parameters [33]. This sample size should, therefore, ensure robust and consistent results while adhering to the principles of the 3Rs (replacement, reduction, and refinement). All efforts were made to minimise animal suffering and reduce waste of edible products by using only the number of carcasses necessary to obtain consistent and reliable results.

After slaughter, the fur, genitals, urinary bladder, and the distal part of the legs were removed. Dissection was carried out according to the guidelines recommended by Blasco et al. [34]. The carcasses were then chilled at 4 °C for 24 h and weighed to determine the chilled carcass weight (CCW), which includes the head, kidneys, and liver, using a technical scale (RUISHAN High Precision Balance 0.01 g RD5002 Shanghai, China). The commercial dressing percentage (CDP) was calculated as the ratio of CCW to SW. Subsequently, the head and internal edible organs were removed, and each carcass was dissected into four main sections. Each section was weighed, and its proportion relative to the total carcass weight was calculated, including fore part yield (FP), thorax yield (TP), loin yield (LP), and hind part yield (HP).

The *Longissimus thoracis et lumborum* (LTL) muscle was excised from both sides of the carcass, carefully trimmed of external fat and epimysium connective tissue, and immediately analysed for pH and colour measurements.

Following dissection, ground meat samples were obtained from different anatomical regions of each carcass using refrigerated mincers (SIRMAN Model TC22, Barcelona, Spain). To prevent overheating of the meat during mincing, the entire meat sample from each carcass was divided into two portions and processed separately, then mixed together before being divided into subsamples for the various analyses. Meat samples were used to assess water-holding capacity (WHC) and cooking loss. The remaining ground meat was stored in Falcon™ plastic tubes (Corning Inc., Corning, NY, USA) at −80 °C in an ultra-low temperature freezer (Thermo Scientific™, Forma 900 Series, Waltham, MA, USA) for subsequent analysis of proximate composition, lipid oxidation, and fatty acid profile. Additionally, leg meat samples were utilised for sensory evaluation by a trained panel to investigate potential nutritional and organoleptic differences related to dietary treatments.

### 2.3. Physical Analyses on Longissimus Thoracis Et Lumborum

#### 2.3.1. pH

The pH was measured 24 h post-mortem by direct insertion into the chilled LTL samples following the insertion protocol described by Pla et al. [35]. Measurements were taken using a portable pH meter (pH/M201, Radiometer Analytical, Villeurbanne, France) equipped with a pHC3031-9 spear-shaped electrode. The device was calibrated with pH 7.01 and pH 4.01 buffer solutions, and the final value was obtained as the mean of two measurements.

#### 2.3.2. Colour

The colour of the chilled LTL muscle was recorded 24 h post-mortem using the CIELAB colour space system, which quantifies lightness (L*), redness (a*), and yellowness (b*). A Konica Minolta CM-3600D spectrophotometer (Sensing, Inc., Osaka, Japan) was employed for the analysis, with a D65 illuminant (6504 °K, daylight). Prior to measurement, the device was calibrated using a standard white reference tile to ensure accuracy.

To standardise the assessment, the muscle was cut longitudinally and exposed to air for 30 min, allowing the blooming effect to stabilise pigment oxidation and enhance colour uniformity. The final colour values were calculated as the mean of four measurements per sample. The CM-3600D spectrophotometer operates with d/8° viewing geometry, which enhances measurement precision, particularly for highly reflective surfaces, such as rabbit meat. This configuration ensures a more reliable reading by reducing the influence of surface gloss. In addition, Chrome, which represents the saturation or intensity of colour and Hue (H°), which indicates the dominant colour tone [36], were calculated using the following equations:

$$Chrome\;\left(C*\right)=\sqrt{{a*}^{2}+{b*}^{2}}$$

$$Hue\;\left(H{}^{\circ}\right)=arctang\left(\frac{b}{a}\right)*57.29$$

The colour differences between groups (ΔE) were calculated as following:

$$\Delta E-=\sqrt{{\left(\Delta L\right)}^{2}+}{\left(\Delta a*\right)}^{2}+{\left(\Delta b*\right)}^{2}$$

where Δ represents the difference in colour parameter between the groups (C vs. L2.5%); (C vs. L5%) and (L2.5% vs. L5%). Differences in perceivable colour can be analytically classified as very distinct (ΔE > 3), distinct (1.5 < ΔE ≤ 3), and small (ΔE ≤ 1.5) as reported in Pathare et al. [36]. Furthermore, reflectance in the visible spectrum was recorded from 360 to 740 nm, and the reflectance ratio at 580 nm/630 nm was calculated to assess colour oxidation. The method used for colour determination was described in detail by Ripoll et al. [37].

#### 2.3.3. Water-Holding Capacity and Cooking Loss

The WHC was determined using 15 g of LTL minced with 15 mL of a salt solution (0.6 M of NaCl) for two minutes, according to the method of Bowker and Zhuang [38]. After two minutes, the mixture was kept at 4 °C for an additional time of 15 min. The mixture was then shaken and centrifuged at 5000 rpm for 15 min. The WHC was calculated using the following formula:

$$WHC\;\left(\%\right)=\frac{\left(0.6\;M\;NaCl\;added-supernatant\;volume\right)}{Sample\;weight}\times 100$$

Cooking loss (CL) was determined according to the method described by Honikel et al. [39]. Uniform meat samples were initially weighed and placed in heat-resistant plastic bags suitable for cooking. The samples were then submerged in a water bath at 85 °C and heated for approximately 25 min until they reached an internal temperature of 75 °C, monitored with a thermocouple. After cooking, the bags were cooled under running tap water, and the meat was patted dry with paper towels. CL was determined as the percentage difference between the samples’ initial and final weights.

### 2.4. Chemical Analyses on Minced Meat from Different Regions of Carcass

#### 2.4.1. Proximate Composition

Proximate composition (dry matter, ash, total fat and crude protein) was measured in duplicate on minced meat samples from the thorax, loin, forearm and leg of each rabbit, according to AOAC methods [40]. Dry matter (DM) was determined by heat-drying the samples in an oven at 105 °C for 24 h (method 934.01). Ash content was determined by incineration in a muffle furnace at 550 °C for 5 h (method 942.05). Total fat was determined using the Soxhlet (method 920.39). Crude protein (CP) content (N × 6.25) was measured using the Kjeldahl method (method 984.13), with the Tecator Digestion System and Kjeltec Auto 1030 Analyzer (Tecator Apeldoorn, Hoganas, Sweden). All data were expressed as a percentage of meat.

#### 2.4.2. Determination of Fatty Acid Profile

Fatty acid methyl esters (FAMEs) were quantified on minced meat samples from thorax, loin, forearm and leg [41]. Meat lipids were extracted in duplicate according to Folch et al. [42] method. The meat lipids were methylated according to the IUPAC method [43] by adding a concentrated solution of KOH (2 N) in methanol. FAMEs were then quantified using a gas-chromatograph (GC 6890N Agilent, Inc., Santa Clara, CA, USA) equipped with a flame ionisation detector (FID), and a CP-Sil88-fused silica capillary column (100 m 0.25 mm (internal diameter) with 0.2 μm film thickness; Agilent Technologies, Inc., Santa Clara, CA, USA) was used. Fatty acids extraction, methylation and gas chromatograph conditions were performed as reported by Failla et al. [44]. Internal standard C19:0 was added to the samples before the fatty acid extraction to perform recovery. Fatty acids methyl esters were identified by comparing the peaks retention time of each compound with standard peaks: Supelco mix 37, CLA mix, 22:4-n-6, 22:5-n-3 DPA (Sigma-Aldrich Merck, Darmstadt, Germany), and a mixture branched chain of BR2 and BR4 (Larodan, Solna, Sweden). The FAME and different classes of fatty acids, including saturated (SFA), monounsaturated (MUFA), and polyunsaturated (PUFA) fatty acids, were expressed as a percentage of the total FAME. Only FAs accounting for more than 0.1% of the total FAME were included in the results with the exception for EPA. Additionally, individual n-3 PUFA fatty acids were quantified in mg per 100 g of meat.

The thrombogenic index (TI) and atherogenic index (AI) were calculated following the equation proposed by Ulbricht and Southgate [45], and peroxidability index (PI) was calculated according to Arakawa and Sagai [46]:

AI = (12:0 + 4 × 14:0 + 16:0)/(∑MUFA + ∑PUFA);

TI = (14:0 + 16:0 + 18:0)/(0.5 × ∑MUFA + 0.5 × ∑n-6 PUFA + 3 × ∑n-3PUFA + n-3/n-6);

PI = (0.025 × ∑monoenoic acids%) + (∑dienoi acids%) + (2 × ∑trienoic acids%) + (4 × ∑tetraenoic acids%) + (6 × ∑pentaenoic acids%) + (8 × ∑hexaenoic acids%).

Fatty acids were also analysed in pellet samples using the same method and expressed as a percentage.

#### 2.4.3. Thiobarbituric Acid Reactive Substance Assay (TBARS)

Lipid oxidation was assessed using the TBARS assay, with malondialdehyde (MDA) serving as the reference compound. A 2.5 g meat sample was homogenised with water and trichloroacetic acid using Ultraturrx IKA (T25 Digital Ultraturrax, IKA, Staufen, Germany). Homogenisation was carried out at 8000 rpm in two 15-s intervals using an ice bath to prevent overheating, followed by centrifugation to obtain the supernatant. This supernatant was then incubated at 80 °C for 30 min with 0.28% thiobarbituric acid (TBA), facilitating the formation of the MDA-TBA complex. After cooling, 10 μL of the mixture was injected into an HPLC system (Alliance 2695, Waters Corporation, Framingham, MA, USA) fitted with a C18 reverse-phase column. The TBARS concentration was determined as mg of MDA per kg of meat, in accordance with the method detailed by Valerio et al. [41].

### 2.5. Sensory Analysis

The sensory test was conducted at the Department LR13AGR02, Mateur-Higher School of Agriculture, University of Carthage (Tunisia), on thigh meat samples collected during dissection and frozen at −18 °C until analysis.

A sensory panel test consisted of 15 trained individuals aged 25 to 45 years with the same gender ratio, who evaluated the meat’s texture, odour/smell, and colour using a scale from 1 (not acceptable) to 10 (extremely acceptable).

The panellists, with prior experience in meat sensory evaluation, participated in continuous periodic training programs to maintain and update their evaluation skills. For the present study, an additional specific training cycle of approximately 4 h, subdivided into 4 sections, was conducted. This training included both theoretical and practical sessions focused on rabbit meat sensory attributes. The training involved sample evaluation exercises to improve the panellists’ ability to recognise key attributes, such as tenderness, juiciness, flavour, and overall acceptability, as well as sessions aimed at standardising the sensory vocabulary used across panellists and enhancing their specificity and discrimination abilities. Training procedures were conducted following ISO 8586:2012 guidelines [47].

Before analysis, the samples were thawed at 4 °C and cooked in an oven at 180 °C until reaching a core temperature of 75 °C. Three cooked thighs for the group were used for each session, divided into 15 equal-sized portions, ensuring uniform meat samples. The portions were wrapped in aluminium foil to retain aroma and heat and were coded with randomly assigned three-digit numbers. The samples were analysed in five different sessions. The data were recorded individually for panellists, sessions and dietary groups.

### 2.6. Statistical Analysis

The effect of dietary supplementation was analysed on production performance, carcass yield, meat quality, FA profile and sensory test of rabbit, using a one-way analysis of variance ANOVA method, with GLM procedure (SAS, 2003, Institute Inc., Cary, NC, USA). Differences among groups were tested by the Tukey’s test. A *p*-value of *p* < 0.05 was considered significant for all measurements. The values were presented as mean with standard error (mean ± SD).

Additionally, the reliability of the sensory evaluation was assessed through distributional analysis and box plot visualisation for each sensory attribute. Descriptive statistics (mean, and standard deviation) were computed to characterise the dispersion of the sensory scores. Score distributions and data normality were further evaluated using histograms, as illustrated in Figures S1 and S2.

Finally, an unsupervised multivariate Principal Components Analysis (PCA) was performed on the qualitative meat data to assess the impact of the main variables on the effect of EL supplementation in the animals’ diet. To standardise the analysed data, which exhibited a wide range of values, a logarithmic transformation was applied Y = log (datum + 1) where Y was the transformed value. This transformation was applied to reduce variability and improve data distribution, making them more suitable for statistical analysis [44]. The results are presented through the distribution score and the loading plot.

## 3. Results and Discussion

Since the inclusion of EL required adjustments to the composition of the basal diet, as reported in Table 1, most notably a reduction in barley content, special attention was paid in the discussion to the potential influence of these compositional changes, beyond the targeted increase in n-3 PUFA levels.

### 3.1. Productive Parameters and Carcass Traits

**Figure 1:**
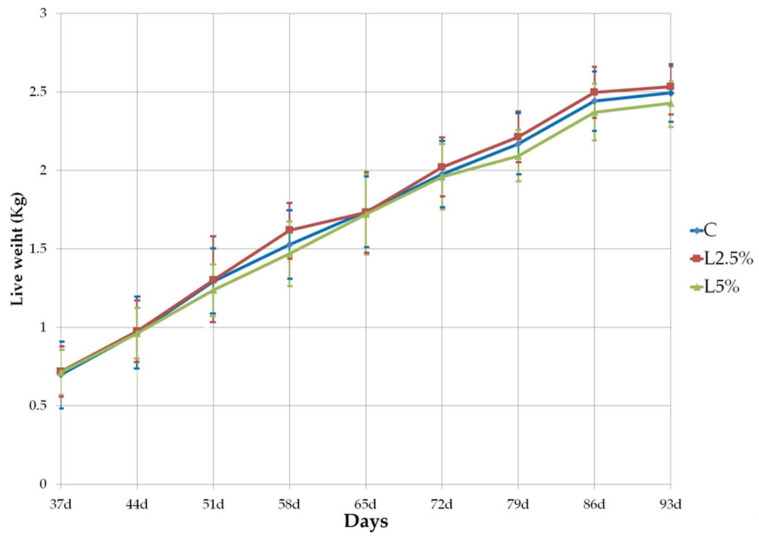
Rabbits’ body weight evolution during fattening period in control group (C), group supplemented with 2.5% of extruded linseed (L2.5%), and group supplemented with 5% of EL (L5%).

During the 8-week trial, dietary treatments had no significant effect on the mortality rate, which averaged approximately 7% across the three groups. The inclusion of EL in the diet did not significantly influence the rabbits’ body weight (*p* > 0.05), with weight increasing similarly across all groups (Figure 1). By the end of the experiment, weight gain accounted for 72.6 ± 7.58%, 71.8 ± 7.09%, and 70.5 ± 7.83% of the initial live weight (LWi at weaning) in the C, L2.5%, and L5% groups, respectively, with no significant differences observed between groups.

The comparative analysis of live performance and carcass characteristics among rabbits fed the two different levels of EL is presented in Table 2. Both LWi and SW were statistically similar across all groups, with mean values of 703.7 g and 2486.8 g, respectively. During the growth stage, the L5% group exhibited a significantly low ADG (30.8 g) compared to the other two groups, which averaged 32.2 g. Daily feed intake (DFI) was 113.6 g on average, with no significant differences observed among groups, resulting in an overall good feed conversion ratio of 3.58. The CCW was not significantly affected by EL supplementation, with an average value of 1490.4 g. However, the CDP significantly improved with EL supplementation (*p* < 0.05), reaching 61.7 ± 2.32% in the L5% group compared to 58.8 ± 2.51% in the C group.

The study evaluated the effects of two different levels of EL on chilled carcass weight (Table 3). The percentage of edible internal organs, including the lungs, liver, and kidneys, remained consistent across groups (*p* = 0.851), with an average value of 5.97%. Similarly, the percentage of head weight showed no significant differences among groups, with a mean value of 9.44%. As expected, no significant differences were observed in the distribution of carcass portions among groups. On average, the FP accounted for 12.61%, the TP for 15.36%, the LP for 20.58%, and the HP for 31.24% of the CCW. In this experiment, the group supplemented with 5% EL exhibited a lower slaughter weight, although the difference was not statistically significant compared to the other groups. This reduction in slaughter weight did not lead to any significant variations in overall carcass weight or the relative proportions of its components.

#### Evaluation of Productive Parameters

The significant reduction in ADG observed in the L5% group may be attributed to multiple factors. Although the extrusion process applied to flaxseed effectively reduces most anti-nutritional compounds, it does not completely eliminate them. Residual levels of substances, such as phytic acid and mucilage, may reduce nutrient digestion and absorption [14,18,19]. Moreover, while a 5% inclusion level of extruded linseed (EL) is generally considered moderate [23], prolonged exposure throughout the entire fattening period could lead to cumulative negative effects [19,48]. Ibrahim et al. [29] reported that higher dietary inclusion levels of flaxseed (6–8%) significantly reduced nutrient digestibility in rabbits, primarily due to the high mucilage content, which increases intestinal viscosity and disrupts nutrient absorption [20,48]. Although feed intake did not differ significantly among the groups in the present study, it is plausible that a slight reduction in digestibility contributed to the lower ADG observed in the L5% group. It should also be noted that technological processing, cultivar characteristics, and storage conditions can influence the residual levels of anti-nutritional factors in flaxseed [14,18,19], potentially affecting growth performance.

The significantly higher carcass yield observed in the L5% group, despite similar carcass weights across groups, could be explained by a reduction in visceral fat, which is removed along with the digestive tract during slaughter. Supporting this hypothesis, Peiretti et al. [49] reported a higher carcass yield in rabbits fed a diet enriched with 8% linseed compared to the control group. Although visceral organ and perivisceral fat weights were not recorded in the present study, evidence from the literature helps to interpret this result. Gugołek et al. [50], in a review evaluating diets with different fat supplementations in rabbits, observed a reduction in perivisceral fat deposition associated with higher dietary PUFA content. Similarly, Yonkova et al. [51] highlighted that variations in dietary fatty acid composition can influence lipogenesis and fat deposition. Moreover, although both diets in this experiment were formulated to be isoenergetic, their energy sources differed; the control diet had a higher proportion of NFE, whereas the L5% diet contained more total fat, predominantly composed by PUFA, as shown in Table 1. It is well established that increased PUFA intake reduces de novo lipogenesis compared to diets rich in carbohydrates or saturated fats [52]. Further supporting this interpretation, Ibrahim et al. [29] also reported a significant reduction in abdominal fat in rabbits fed diets with linseed inclusion levels above 4%.

Finally, consistent with our findings, several studies have indicated that extruded linseed supplementation does not significantly affect certain live performance parameters [11,16] or carcass composition [10,16,49], even at higher inclusion levels [53]. This underscores the fact that carcass component weights and proportions are primarily influenced by factors such as slaughter weight, animal age, sex, and breed of the animals [54].

### 3.2. Physical Characteristics of LTL Muscle of Rabbit

The analysis of the physical characteristics of the LTL muscle rabbits subjected to different dietary treatments (C, L2.5%, L5%), revealed notable effects on WHC, colour parameters, and oxidative stability (Table 4). pH values that range from 5.56 to 5.77 did not exhibit significant differences among groups (*p* = 0.55).

WHC significantly decreased (*p* = 0.03) in the L5% group (68.3% vs. 85.0% for the L5% and C groups, respectively), suggesting that higher EL supplementation adversely affected the muscle’s water retention capacity. However, cooking loss did not differ significantly among the groups (*p* = 0.64). In addition, EL supplementation significantly influenced meat colour. The L5% group had significantly lower (*p* = 0.011) lightness (L*), compared to the other groups (55.61 vs. 57.34 on average for the others). The L5% group also exhibited significantly higher (*p* < 0.05) redness (a*) and yellowness (b*) (1.30 and 6.23, respectively, for a* and b*), compared to the other groups (0.71 on average for a* and 4.67 on average for b*). Similarly, chrome was significantly higher (*p* = 0.019) in the L5% group, indicating a positive shift toward a more intense colour. However, the hue angle did not differ significantly among groups. Therefore, even though the colour intensity changed, the overall tone remained constant.

The colour differences (ΔE) between groups were calculated to assess the perceptibility of changes in meat colour due to dietary treatments. The comparison between C and L2.5% showed a ΔE value of 0.25, indicating no perceptible colour difference. In contrast, when comparing the L5% group with C and L2.5% groups, ΔE values of 2.43 and 2.37 were recorded, respectively.

The 580 nm/630 nm ratio, an indicator of meat oxidation [55], showed significant differences (*p* = 0.04). The L5% group had a higher value (0.91) compared to the control (0.79), but did not differ significantly from the L2.5% group.

#### Evaluation of Physical Characteristics of LTL

The observed decrease in WHC in rabbit meat from the L5% group compared to C group may be attributed to both more intense post-mortem glycolysis, indicated by a lower ultimate pH, although not statistically significant, and oxidative modifications of muscle proteins [41]. Rabbits in this group received a higher dietary intake of n-3 polyunsaturated fatty acids (PUFAs), which are more susceptible to lipid peroxidation [44]. This peroxidation generates reactive oxygen species that can oxidise muscle proteins [56], leading to structural damage and reduced water-binding capacity. Oxidative stress may also affect sarcoplasmic proteins, which play a critical role in water retention [38], further contributing to the observed reduction in WHC due to their diminished ability to retain water. These findings are consistent with studies showing that dietary linseed supplementation in rabbits can increase oxidation [11,15,23,56]. However, other authors have reported no significant differences in WHC between control and linseed-supplemented groups [57]. Regarding cooking loss, our findings are in line with previous studies reporting no significant differences among different levels of linseed supplementation. For instance, Bianchi et al. [57], who tested linseed inclusion at 3%, 6%, and 9%, observed cooking loss values lower than those recorded in the present study. Conversely, Kahan et al. [58] reported higher WHC and cooking loss values compared to our results, despite finding no statistically significant differences among dietary treatments.

Colour is a key attribute influencing consumer preference [2]. The effects observed in our study following EL supplementation are consistent with the findings of Eiben et al. [59], who demonstrated that replacing 4% of sunflower oil with linseed oil in rabbit diets resulted in lower lightness (L*) values in the *longissimus thoracis* muscle (from 53.4 to 49.7), along with changes in redness (a*) from −0.39 to 1.38, and yellowness (b*) from 4.36 to 5.84. The lightness and redness values reported by Eiben et al. [59], and later referenced by Fehri et al. [16] and Du et al. [60], are generally lower than those observed in our study. Similar colour values, though without significant differences attributable to linseed supplementation, were also reported by Molette et al. [61], who found a higher average a* value (4.4 on average), and by Benatmane et al. [11], who recorded a lower average b* value (2.7 on average). Variability in a* and b* values across studies is often attributed to differences in colourimeter geometry or the use of distinct measurement systems [16]. Nevertheless, post-mortem biochemical processes remain the main drivers of meat colour development and stability [51].

Although the differences observed in the 580 nm/630 nm absorbance ratio among groups might be linked to the higher oxidative susceptibility of n-3 PUFAs, it is not possible to directly compare this ratio with values reported in the existing literature, due to a lack of standardised reference data. However, it is well established that these absorbance bands are highly correlated with myoglobin oxidation and, consequently, with visual meat colour stability. Particularly, Cifuni et al. [62] demonstrated that the 580 nm and 630 nm wavelengths are strongly associated with oxidative changes in rabbit meat. These spectral shifts are therefore considered reliable indicators of pigment oxidation, supporting the idea that dietary enrichment with highly unsaturated fatty acids may affect meat colour via increased oxidative processes.

The ΔE results suggest that the inclusion of 5% extruded linseed induced a colour change approaching the threshold for distinct perceptibility (ΔE > 2), although it remained below the conventional threshold for very distinct differences (ΔE > 3). Only a few studies have evaluated the impact of dietary linseed inclusion on rabbit meat colour using ΔE calculations. For example, Ibrahim et al. [29] reported that supplementation with 8% whole flaxseed resulted in a noticeable colour change, with a ΔE value around 3 when compared to rabbits not receiving linseed supplementation, indicating a perceptible difference in meat colour. However, it is important to note that linseed supplementation does not consistently lead to perceptible colour differences in rabbit meat, as reported previously [11,16,57]. When no significant changes are observed in the L, a*, and b* parameters, ΔE values greater than 2 are unlikely to be recorded. Consequently, detectable colour differences are typically associated with significant variations in the individual colourimetric coordinates.

### 3.3. Proximate Composition and Fatty Acids Profile in Rabbit Meat

The inclusion of EL in the diet resulted in a higher fat accumulation (+1.4%), when comparing the C group with the group receiving 5% EL (Table 5), which was reflected in a higher percentage of dry matter; while the other components (protein and ash%) did not differ significantly among the three groups.

The effects of dietary supplementation with EL on the FA composition of rabbit meat were evaluated in Table 6. The results showed a significantly lower percentage in ∑SFA (*p* < 0.001) for the L5% group (33.06%) compared to the C group (35.78%). This difference was mainly attributable to palmitic acid (16:0), the predominant saturated fatty acid in rabbit meat, which ranged from 22.26% in the L5% group to 24.62% in the C group. The other SFAs did not differ significantly among the groups. Apart from stearic acid (18:0), which averaged 7.82% across all groups, the remaining SFA listed in Table 6 did not exceed 2%.

The ∑MUFA remained consistent among all groups (*p* = 0.207), ranging from 22.13% to 23.71%. No significant differences were observed for individual MUFA. Oleic acid (18:1 cis-9) was the most abundant, with 18.21% as the average of the three groups. All other MUFAs individually contributed less than 2%.

In contrast, ∑PUFA increased slightly with EL supplementation, with the highest value recorded in the L5% group (40.31%), which differed significantly from the C group (*p* = 0.011). These differences were primarily due to an increase in n-3 PUFA, as the meat of rabbits in the L5% group showed a marked enrichment in these fatty acids compared to the C group (9.11% vs. 3.18%, respectively), attributable to the supplementation with EL.

Significant differences were also recorded for total n-6 PUFA between C and L5% groups (*p* = 0.004), as linseed inclusion partially replaced barley in the diet, a cereal rich in n-6 fatty acids (see Table 1).

Among the n-6 PUFA, LA (18:2 n-6) was the most abundant, showing no significant differences among groups (*p* = 0.316), with a mean value of 28.01%. In contrast, significant differences (*p* < 0.05) were observed for long-chain n-6 PUFA (n-6 LC-PUFA). Specifically, eicosadienoic acid (20:2 n-6) decreased from 0.27% in the C group to 0.21% in L5%, and arachidonic acid (20:4 n-6), the second most abundant n-6 PUFA in rabbit meat, decreased from 4.46% to 3.25% between C and L5% groups, respectively.

The experimental intervention, i.e., dietary inclusion of n-3 PUFA via EL, significantly affected (*p* < 0.001) the composition of ALA (18:3 n-3), the predominant n-3 PUFA in rabbit meat. Its content increased with the linseed level in the diet, from 2.27% in the C group, to 4.13% in L2.5%, and up to 7.42% in L5%, with all three groups differing significantly from one another. This represented an approximate +45 percentage point (p.p.) increase among groups.

Significant differences (*p* < 0.05) were also found among the three groups for long-chain n-3 PUFA (n-3 LC-PUFA), except for EPA (20:5 n-3), which showed no significant differences, and had an overall mean value of 0.06%. The other two n-3 LC-PUFAs increased with the level of EL in diet, particularly DPA (22:5 n-3), which showed a value of 0.47%, 0.68%, and 1.11% in C, L2.5%, and L5% groups, respectively. The most pronounced increase was observed between the L2.5% and L5% groups (+38 p.p.).

The proportion of n-3 PUFAs in meat affects the values of certain nutritional indices, as reported in Table 7. The n-6/n-3 ratio ranged from 10.79 in group C to 3.34 in the L5% group. The L2.5% group showed an intermediate value, which nevertheless differed significantly (*p* < 0.001) from those of the other two groups. The SFA/PUFA ratio exhibited significant differences (*p* = 0.004), only between the C and L5% groups, with a difference of −11 p.p. The lowest values for both the TI and AI were reported in the L5% group (*p* < 0.01) compared to the other two groups. In detail TI was 0.576 for L5% vs. 0.827 as the average of the other two groups, and the AI index 0.451 for L5% vs. 0.504 as the average of the other two. For PI index, only group C differed significantly from L5% (+11 p.p.). The amount of malondialdehyde in the meat was analysed to assess the extent of lipid oxidation, the two EL-supplemented groups showed TBARS values that did not differ significantly from each other but were significantly higher than those of the C group (0.057 mg/kg as the average of the EL-supplemented groups vs. 0.045 mg/kg in the C group).

**Figure 2:**
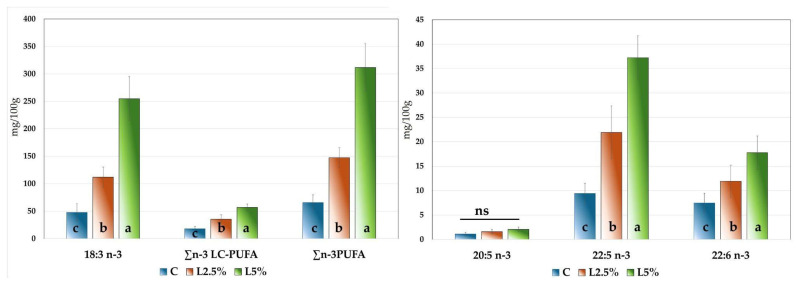
Composition of omega-3 polyunsaturated fatty acids (n-3 PUFA), including long-chain n-3 LC-PUFA, α linolenic acid (18:3 n-3, ALA), eicosapentaenoic acid (20:5 n-3, EPA), docosapentaenoic acid (22:5 n-3, DPA), and docosahexaenoic acid (22:6 n-3, DHA), expressed as mg per 100 g of minced rabbit meat from animals fed different diets. C = control group; L2.5% = group supplemented with 2.5% EL; L5% = group supplemented with 5% EL. Different superscript letters on the histograms for the same fatty acid indicate significant differences (*p* < 0.05), and ns = not significant for *p* > 0.05.

As shown in Figure 2, where n-3 PUFAs are expressed in mg per 100 g of meat, the groups supplemented with EL exhibited significantly higher levels of alpha-linolenic acid (18:3 n-3), with values differing significantly among the three groups (*p* < 0.001). The concentration of this fatty acid increased from 47.88 mg/100 g in the C group to 111.78 mg/100 g in the L2.5% group, reaching a maximum of 254.76 mg/100 g in the L5% group.

It is worth noting that the meat from the L5% group was also significantly fattier than that of the other groups (Table 5). The concentrations of n-3 LC-PUFAs, expressed in mg per 100 g of meat, followed a similar pattern to that observed in the percentage distribution. EPA (20:5 n-3) did not show significant differences among groups, with an average concentration of 1.59 mg/100 g. In contrast, both DPA (22:5 n-3) and DHA (22:6 n-3) increased significantly (*p* < 0.001) from group C to group L5%. Specifically, DPA rose from 9.41 mg/100 g in the C group to 37.22 mg/100 g in the L5% group, while DHA increased from 7.45 mg/100 g to 17.37 mg/100 g, respectively.

Notably, in the control group, DPA represented 52% of the total n-3 LC-PUFAs, whereas in the supplemented groups this proportion increased to 62%, highlighting the significant impact of flaxseed supplementation on the long-chain fatty acid profile of rabbit meat.

#### Evaluation of Proximate Composition and Fatty Acid Profile in Relation to Dietary and Metabolic Factors

As shown in Table 1, although the diets were isoenergetic, the EL-supplemented groups showed a higher lipid content, which may contribute to greater fat accumulation in the meat [63]. Several studies [10,16,40,57] have reported lower fat percentages, with no significant differences attributable to linseed supplementation. However, in those studies, chemical analyses were performed on the LTL muscle, which is typically the leanest muscle in the carcass. In contrast, the samples analysed in the present study were derived from mixed meat portions taken from various carcass regions. Other authors have even reported higher fat values in thigh meat than those observed in our study, particularly when animals were slaughtered at older ages or reached heavier final weights [29,58].

Rabbit meat is naturally characterised by a relatively high PUFA content, ranging from 35% to 42%, levels typically found only in game meats [7,64]. In contrast, MUFA levels remain low, even in rabbits fed diets rich in oleic acid [9,16]. When compared to other types of meat, rabbit meat shows a similar SFA content (approximately 38.5%) to pork, beef, and veal, but higher than that of chicken (29.5%) [6,65].

However, recent feeding strategies aimed at improving the health value of rabbit meat have led to modifications in dietary composition. Recommendations for rabbit nutrition emphasise a higher fibre intake [66], which has promoted a shift from concentrated feeds to greater inclusion of alfalfa and oilseeds. This approach, as shown by multiple studies [11,17,57,58,67], has successfully lowered the SFA content of rabbit meat while increasing its PUFA proportion. Nevertheless, this reduction has mainly affected stearic acid (18:0), while palmitic acid (16:0) remained at levels comparable to those observed in other meats [8,10,15,57]. In our study, the inclusion of extruded linseed (EL) effectively reduced palmitic acid levels in meat, confirming results reported by other authors [17,49,57,68], and contributing to a more favourable lipid profile for consumers [69].

However, particular attention should be given to the quality of PUFA, promoting the accumulation of n-3 PUFA in meat. Diets enriched with linseed effectively address this nutritional goal [12].

Although the primary aim of our work was to enhance n-3 PUFA content, it is important to underline that EL supplementation induced broader compositional changes. In the EL groups, energy derived from NFE in the control diet was partially replaced by lipids, modifying the balance of SFA, MUFA, and PUFA. These adjustments, adopted in many experiments to correct integration of EL in the diet [15,17,29,49,58], must be considered when interpreting the effects of EL supplementation, as differences observed among groups cannot be attributed solely to increased n-3 intake.

In this context, particular attention should be given not just to the total PUFA content, but to its quality. Linseed-based diets effectively promote ALA (18:3n-3) enrichment in rabbit meat, as confirmed by the close alignment between our data and predictive models proposed by Petracci et al. [23]. However, the increase in ALA does not follow a strictly linear response to the inclusion level; it is also affected by the duration of supplementation, as shown by Matics et al. [17]. Their study demonstrated a progressive rise in ALA and n-3 LC-PUFAs with longer feeding periods.

Rabbits, like all mammals, are unable to synthesise essential fatty acids de novo, such as linoleic acid (LA, 18:2n-6) and alpha-linolenic acid (ALA, 18:3n-3), which must be supplied through the diet [70]. Once ingested, PUFAs are emulsified in the intestinal lumen and incorporated into chylomicrons following enterocyte absorption. They are then transported via the lymphatic and circulatory systems to various tissues [71].

The balance between n-6 and n-3 fatty acids is critical, as these compounds compete for the same enzymatic pathways (desaturases and elongases) involved in the biosynthesis of LC-PUFAs [72,73]. These reactions form a complex cascade that converts short-chain fatty acids into long- and very-long-chain PUFAs, a process crucial for PUFA accumulation in meat.

The two main precursors, LA and ALA, undergo a sequence of reactions catalysed by desaturases and elongases. Δ6-desaturase is the first enzyme in this cascade, introducing a double bond at the Δ6 position of both LA and ALA. Elongation by ELOVL5 extends the carbon chain from C18 to C20, and Δ5-desaturase subsequently adds another double bond at the Δ5 position, leading to the formation of ARA (20:4n-6) and EPA (20:5n-3) [71,74,75]. To produce DHA (22:6n-3), additional steps are required: further elongation by ELOVL2, a second Δ6-desaturation, and a peroxisomal β-oxidation step that shortens the chain. Alternatively, DHA may be synthesised from DPA via the action of Δ4-desaturase, which introduces an additional double bond [26,27,75].

In rabbits, the enzymatic conversion of ALA to its long-chain derivatives, such as EPA and DHA, is limited due to relatively low Δ6-desaturase activity. As a result, the majority of ALA remains unmetabolised and is directly deposited in muscle tissue, leading to a measurable increase in total n-3 PUFA levels [10]. This metabolic complexity is reflected in our results. While DPA levels increased significantly in both EL-supplemented groups, DHA levels showed only moderate increases. Furthermore, the L2.5% group did not consistently exhibit intermediate values across all measured fatty acids. Notably, its SFA content and levels of arachidonic acid (ARA) were statistically similar to those of the control group. This suggests that the response to EL supplementation depends not only on the intake of omega-3 fatty acids derived from linseed, but also on the relative availability of other dietary components that modulate fatty acid metabolism.

Meat, particularly that rich in PUFAs, such as chicken, rabbit, and fish, is a source of LC-PUFAs, which cannot be obtained from plant-based foods and are essential for various metabolic functions. Jayaprakash et al. [22], in fact, described these compounds as bioactive lipids. Unlike n-6 LC-PUFAs, which are abundantly available in animal-derived products and certain plant seeds, n-3 LC-PUFAs are present in high amounts only in fish and in some algae [25,76]. Meat represents the main dietary source of DPA (22:5 n-3), which accumulates in the tissues of mammals and poultry [21], while fish, although rich in EPA and DHA [24,76], contains comparatively lower levels of DPA [24,25,26]. Although research on the health significance of DPA is limited [26,27], it has been suggested that DPA may play an important role in preventing cardiovascular and metabolic diseases, potentially with greater efficiency than EPA and DHA, due to its higher oxidative stability and consequently greater bioavailability [26].

Western diets typically exhibit an n-6/n-3 ratio more than 10:1, and such elevated ratios are hypothesised to contribute to the development of chronic diseases [72]. A ratio of 4:1 has been associated with a significant reduction in the risk of various metabolic and cardiovascular disorders [67,77]. The World Health Organization (WHO) recommends an intake of LA ranging from 2.5% to 9% of total energy in adults, and a minimum intake of 0.5% to 2% for ALA [78]. The European Food Safety Authority (EFSA), in its 2010 guidelines [79], established reference intake values of 4% of total energy for LA and 0.5% for ALA. Both the WHO and EFSA recommend a minimum daily intake of 250 mg of long-chain n-3 fatty acids.

The SFA/PUFA ratio in rabbits approaches unity, as the high content of PUFA only partially reduces the accumulation of SFA in the meat [65,80]. Excessive dietary intake of SFA is associated with cardiovascular diseases, metabolic disorders, and cancer; therefore, SFA consumption should be limited according to nutritional guidelines issued by health organisations, such as the WHO and EFSA [78,79].

The AI and TI indices, calculated from fatty acid composition, were developed by Ulbricht and Southgate [45]. These indices represent ratios comparing certain SFA to MUFA and PUFA. The AI assigns equal coefficients to MUFA and PUFA but gives a fourfold higher weight to myristic acid due to its strong cholesterol-raising effect, while stearic acid, considered neutral, is excluded from this calculation. A higher AI value indicates that the fatty acid profile of the meat promotes greater cholesterol accumulation. Conversely, a higher TI value suggests that fat has a greater tendency to induce thrombosis, with stearic acid also regarded negatively in this index [45].

In rabbit meat, both indices (AI and TI) are notably lower compared to other meat types [81]. Nevertheless, supplementation with flaxseed further reduces these index values, as reported in some experiments [10,18].

Additionally, PUFAs that are not incorporated into membrane phospholipids may be stored in muscle as triglycerides or catabolised via β-oxidation, particularly in the mitochondria, where they are used to generate energy. Among all fatty acids, n-3 PUFAs are those most readily oxidised in oxidative muscle fibres [44].

The PI, which was associated with the presence of PUFAs in the meat, is characterised by greater instability due to the number of double bonds in FAs. It showed a trend opposite to that of the SFA/PUFA ratio.

The peroxidation index closely correlates with TBARS values, indicating the limited stability of polyunsaturated fatty acids, which increase when animals are supplemented with EL. Additionally, both parameters are consistent with the myoglobin oxidation index (measured as the absorbance ratio at 580 nm/630 nm; see Table 4). The impact of dietary inclusion of linseed on lipid instability has also been highlighted by several authors [10,15,23,56,68]. However, as shown in Figure 2, it is important to highlight that the sum of n-3 PUFAs exceeded 300 mg/100 g of meat, the threshold defined by EFSA [82,83] for a food product to be labelled as a source of omega-3 fatty acids. These findings support the potential of EL-enriched diets to produce functional meat products

### 3.4. Sensory Evaluation

**Figure 3:**
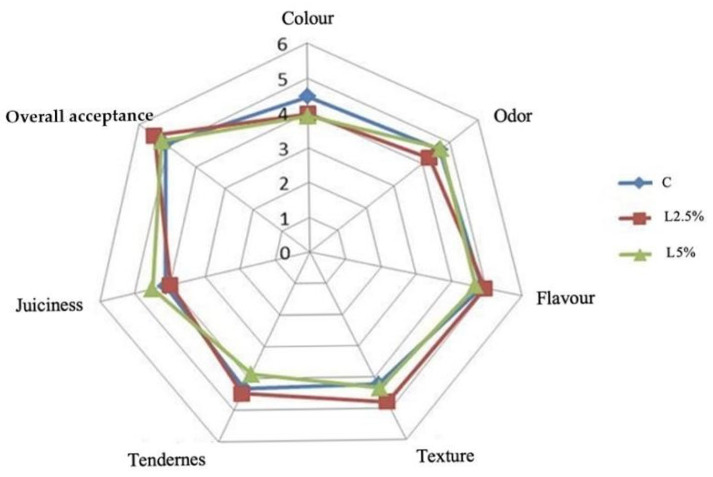
Organoleptic characteristics of rabbit meat fed with different diets. C = control group; L2.5% = group supplemented with 2.5% EL; L5% = group supplemented with 5% EL.

The results of the panel are presented in Figure 3. The data were presented as average scores assigned to each batch by 15 panellists for the different organoleptic characteristics of rabbit meat, comparing samples with and without linseed inclusion. The distribution and box plot of the scores recorded are presented in Supplementary Figures S1 and S2.

Panellists evaluating the sensory aspects of rabbit meat supplemented with EL used different sensory attributes, such as colour, odour, flavour, texture, tenderness, juiciness, and overall acceptance.

These attributes were not significantly affected by the diets (*p* > 0.05). Nevertheless, the C group showed a better trend in colour parameters than the EL supplemented groups (*p* < 0.10); a trend that was also confirmed by instrumental meat colour analysis.

As a primary sensory quality, colour plays a crucial role in consumer perception of food products, influencing their decision to accept or reject a product [2,10]. Our sensory analysis finding was in agreement with some authors [2,84] who reported no significant difference in rabbits fed with different percentages of EL compared to the C group. Overall, the assessors did not find any differences in overall acceptance, even though this result was unexpected because an increase in fatty acids, PUFAs in particular, should influence some characteristics, such as odour and tenderness [84] after the cooking process.

However, the results of Kumar et al. [65] highlight that variations in colour and odour between meat with and without EL supplementation could be attributed to various factors.

### 3.5. Principal Component Analysis (PCA) of the Qualitative Parameters of Meat

**Figure 4:**
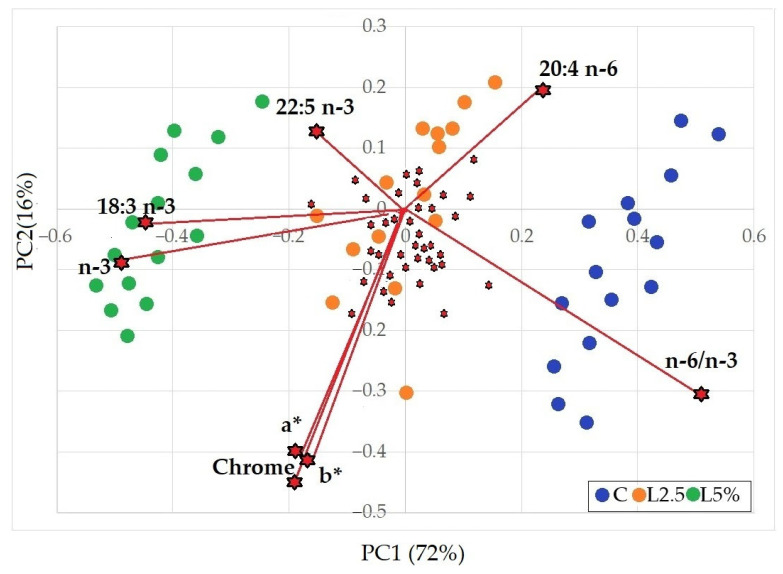
Plot of loading and scores from Principal Component Analysis (PCA) of the main qualitative parameters of rabbit meat fed with different diets. C = control group; L2.5% = group supplemented with 2.5% EL; L5% = group supplemented with 5% EL.

The multivariate PCA analysis, conducted using physicochemical composition and sensory parameters (Figure 4), accounted for 88% of the total variability when considering the first two principal components (PC1 and PC2). The score plot clearly showed a distinct separation among the three dietary groups, with the L2.5% group positioned intermediately between the C and L5% groups.

The loading plot revealed that the n-3 PUFA in the meat, reflecting the main effect of EL inclusion in the diet, contributed the most to the observed variability, along with the n-6/n-3 ratio. This ratio appeared in an adjacent quadrant, as it depends on both PUFA fractions. Near the total n-3 PUFA vector, the 18:3n-3 also showed a strong contribution.

Among the long-chain fatty acids, only 20:4n-6 and 22:5n-3 (DPA) were noteworthy. The former was located in the quadrant opposite to that of n-3 fatty acids and ALA, while DPA, considered, even in the multivariate analysis, a key contributor to the nutritional improvement of rabbit meat from linseed-fed animals, was positioned in the upper section, aligned with the direction of other n-3 FAs and close to the cluster of the L5% group.

Meat colour parameters, particularly a*, b*, and chrome, also accounted for a substantial portion of variability and were positioned opposite to the region where L2.5% group scores were predominantly distributed. The other qualitative parameters considered appeared to have little influence in discriminating among the three groups.

## 4. Conclusions

This study provides evidence that dietary supplementation with EL improves the fatty acid profile of rabbit meat, particularly by increasing the accumulation of n-3 PUFAs, including long-chain derivatives, such as DPA and DHA. Although a moderate reduction in average daily gain was observed at the highest inclusion level (5%), overall productive performance and carcass weight remained unaffected. Moreover, a significant improvement in commercial dressing percentage suggests modifications in lipid metabolism, potentially leading to reduced visceral fat deposition.

Meat quality was influenced in several aspects. EL supplementation resulted in higher intramuscular fat content and alterations in colour parameters, along with significant improvements in key nutritional indices, such as the n-6/n-3 ratio, atherogenic index, and thrombogenic index. However, increased lipid unsaturation was associated with reduced water-holding capacity and greater oxidative susceptibility. Importantly, sensory characteristics were not negatively affected, indicating that the organoleptic profile of the meat was maintained despite compositional changes. It is also noteworthy that the group supplemented with 2.5% EL, despite showing an appreciable improvement in the n-3 PUFA profile, did not differ significantly from the control group in many other meat quality traits. This suggests that the effects of lower inclusion levels may be more limited.

In conclusion, EL supplementation appears to be a promising nutritional strategy to enhance the nutritional value of rabbit meat, particularly through the enrichment of long-chain n-3 PUFAs. Further investigations should focus on elucidating the metabolic pathways involved in fatty acid elongation and deposition and on developing dietary approaches to improve oxidative stability during storage. 

## Figures and Tables

**Table 1:** Feed formulation of the diets, chemical composition (% of dry matter), fatty acid profile (% of total FAME) of the three experimental groups.

Ingredients (%)	C	L2.5%	L5%
Extruded Linseed	0	2.5	5.0
Barley	20.9	19.4	17.9
Soybean flour	12.1	12.1	12.1
Soybean flakes	20.0	20.0	20.0
Soybean meal	8.2	8.2	8.2
Alfalfa	26.8	25.8	24.8
Wheat bran	5.0	5.0	5.0
Vitamin mineral premix	4.0	4.0	4.0
Calcium carbonate	3.0	3.0	3.0
Nutrient composition (% of DM)			
Crude protein (CP)	17.46	17.49	17.53
Ash	6.80	6.78	6.73
Crude Fat (EE)	3.30	3.61	4.03
Crude fibre (CF)	11.40	11.58	11.75
Nitrogen-Free Extractives (NFE)	61.04	60.54	59.96
Crude Cellulose	17.81	17.58	17.54
Calcium (g/100g)	1.85	1.81	1.78
Phosphor (g/100g)	0.6	0.61	0.62
Digestible energy (kcal/kg) *	2101.7	2116.5	2130.3
Fatty acids (% of total FAME)			
16:0	14.17	12.42	11.12
18:0	3.20	3.13	2.31
18:1 n-9	23.16	22.70	21.24
18:2 n-6	51.31	42.76	39.23
18:3 n-3	8.16	18.99	26.10

C = control diet, L2.5% = diet supplemented with 2.5% EL; L5% = diet supplemented with 5% EL; DM = dry matter (88.3 g/100 g on average for the three pellets); FAME = fatty acid methyl esters, * Estimated according to Maertens et al. [30]. Digestible energy (kcal/kg) = −7.54 + (29.71 × CP) + (50.25 × EE) + (23.34 × NFE). 16:0 = palmitic acid, 18:0 = stearic acid, 18:1 n-9 = oleic acid, 18:2 n-6 = linoleic acid (LA), 18:3 n-3 = α linolenic acid (ALA).

**Table 2:** Effect of extruded linseed supplementation on rabbits’ productive performance.

% CCW	C	L2.5%	L5%	*p* Value
LWi (g)	697.4 ± 91.7	716.1 ± 81.7	712.5 ± 69.4	0.754
SW (g)	2495.1 ± 93.6	2538.4 ± 128.8	2427.1 ± 117.4	0.285
ADG (g)	32.1 ± 0.82 ^a^	32.5 ± 0.84 ^a^	30.6 ± 0.97 ^b^	0.038
DFI (g)	114.5 ± 3.8	113.8 ± 3.6	112.6 ± 5.2	0.730
FCR	3.57 ± 0.18	3.50 ± 0.19	3.68 ± 0.23	0.321
CCW (g)	1467.3 ± 52.4	1507.1 ± 54.1	1496.7 ± 34.5	0.542
CDP (%)	58.8 ± 2.01 ^b^	59.4 ± 2.21 ^ab^	61.7 ± 2.12 ^a^	0.041

LWi = rabbits’ weight at 37 days; SW = rabbits’ weight at slaughter; ADG = average daily gain; DFI = average daily feed intake; FCR = feed conversion ratio; CCW = carcass weight; CDP = gain commercial dressing percentage. C = control group; L2.5% = group supplemented with 2.5% of EL; L5% = group supplemented with 5% of EL. Different letters on the same row mean significant differences for *p* < 0.05.

**Table 3:** Effect of extruded linseed supplementation on rabbits’ carcass trait proportions.

% CCW	C	L2.5%	L5%	*p* Value
Head	8.94 ± 0.64	9.67 ± 0.37	9.72 ± 0.44	0.352
Edible offal	6.01 ± 0.87	5.78 ± 0.40	6.12 ± 0.80	0.851
FP	12.57 ± 0.60	12.68 ± 0.45	12.58 ± 0.97	0.452
TP	15.58 ± 3.11	14.08 ± 1.11	16.44 ± 3.12	0.150
LP	20.56 ± 1.86	20.88 ± 1.40	20.31 ± 1.50	0.592
HP	31.22 ± 1.12	31.99 ± 0.67	30.53 ± 1.54	0.374

CCW = chilled carcass weight; Edible offal = lung, heart, liver and kidneys%; FP = fore part yield%; TP = thorax yield%; LP = loin yield%; HP = hind part yield%. C = control group; L2.5% = group supplemented with 2.5% EL; L5% = group supplemented with 5% EL. Different letters on the same row mean significant differences for *p* < 0.05.

**Table 4:** Physical-chemical characteristics of *Longissimus thoracis et lumborum* muscle in rabbits feed different diets.

	C	L2.5%	L5%	*p*-Value
pH	5.77 ± 0.22	5.67 ± 0.08	5.56 ± 0.29	0.553
WHC (%)	85.0 ± 5.15 ^a^	83.3 ± 5.77 ^ab^	68.3 ± 7.63 ^b^	0.031
Cooking loss (%)	21.15 ± 2.25	20.20 ± 2.90	22.25 ± 3.71	0.683
L*	57.31 ± 1.36 ^a^	57.37 ± 1.38 ^a^	55.61 ± 1.23 ^b^	0.011
a*	0.77 ± 0.61 ^b^	0.66 ± 0.32 ^b^	1.30 ± 0.52 ^a^	0.033
b*	4.57 ± 0.98 ^b^	4.78 ± 1.09 ^b^	6.23 ± 1.41 ^a^	0.024
Chrome	4.65 ± 1.05 ^b^	4.83 ± 1.21 ^b^	6.38 ± 1.44 ^a^	0.019
Hue	81.07 ± 3.94	81.81 ± 3.21	78.05 ± 4.90	0.282
580 nm/630 nm	0.79 ± 0.04 ^b^	0.81 ± 0.05 ^ab^	0.84 ± 0.03 ^a^	0.042

WHC = water-holding capacity, L* = lightness; a* = redness; b* = yellowness; 580 nm/630 nm = oxidation index. C = control group; L2.5% = group supplemented with 2.5% EL; L5% = group supplemented with 5% EL. Different letters on the same row mean significant differences for *p* < 0.05.

**Table 5:** Proximate composition of minced meat samples of rabbits fed different diets.

%	C	L2.5%	L5%	*p*-Value
Dry Matter	24.81 ± 0.59 ^b^	25.10 ± 0.43 ^ab^	25.51 ± 0.45 ^a^	0.010
Ash	1.16 ± 0.07	1.13 ± 0.04	1.11 ± 0.05	0.204
Total fat	2.34 ± 0.30 ^c^	3.04 ± 0.38 ^b^	3.76 ± 0.32 ^a^	<0.001
Protein	20.94 ± 0.56	20.57 ± 0.52	20.35 ± 0.21	0.235

C = control group; L2.5% = group supplemented with 2.5% of extruded linseed; L5% = group supplemented with 5% of EL. Different letters on the same row mean significant differences for *p* < 0.05.

**Table 6:** Principal fatty acids and their classification expressed as a percentage of total FAME in the thigh meat of rabbits fed different diets.

FA %	C	L2.5%	L5%	*p*-Value
∑SFA	35.78 ± 2.11 ^a^	35.21 ± 1.18 ^a^	33.06 ± 1.52 ^b^	<0.001
14:0	1.66 ± 0.37	1.71 ± 0.41	1.64 ± 0.28	0.194
15:0	0.43 ± 0.04	0.45 ± 0.03	0.42 ± 0.03	0.877
16:0	24.62 ± 1.88 ^a^	23.88 ± 1.27 ^ab^	22.26 ± 0.91 ^b^	0.002
17:0	0.52 ± 0.04	0.47 ± 0.07	0.46 ± 0.03	0.179
18:0	7.85 ± 0.61	7.98 ± 0.81	7.63 ± 0.78	0.111
∑MUFA	22.99 ± 1.78	22.13 ± 1.66	23.71 ± 1.59	0.207
16:1cis-7	0.31 ± 0.04	0.28 ± 0.05	0.30 ± 0.04	0.225
16:1cis-9	1.83 ± 0.68	1.65 ± 0.44	1.76 ± 0.35	0.354
17:1cis-9	0.15 ± 0.03	0.14 ± 0.02	0.15 ± 0.02	0.321
18:1trans9-11	0.11 ± 0.04	0.13 ± 0.06	0.11 ± 0.03	0.361
18:1cis-9	18.04 ± 1.40	17.46 ± 1.67	19.13 ± 1.61	0.210
18:1cis-11	1.60 ± 0.13	1.51 ± 0.23	1.44 ± 0.16	0.119
22:1cis-11	0.44 ± 0.13	0.44 ± 0.13	0.34 ± 0.14	0.721
∑PUFA	38.42 ± 2.87 ^b^	39.79 ± 1.87 ^ab^	40.31 ± 2.12 ^a^	0.011
18:2n-6 LA	29.01 ± 3.20	28.57 ± 2.30	26.46 ± 2.89	0.316
18:3n-3 ALA	2.27 ± 0.39 ^c^	4.13 ± 0.73 ^b^	7.42 ± 1.28 ^a^	<0.001
20:2n-6	0.27 ± 0.04 ^a^	0.28 ± 0.06 ^a^	0.21 ± 0.03 ^b^	<0.001
20:4n-6 AA	4.46 ± 1.29 ^a^	4.15 ± 1.49 ^ab^	3.25 ± 1.17 ^b^	0.041
20:5n-3 EPA	0.06 ± 0.02	0.06 ± 0.02	0.07 ± 0.02	0.113
22:4n-6	0.59 ± 0.14	0.52 ± 0.21	0.51 ± 0.18	0.140
22:5n-3 DPA	0.47 ± 0.15 ^c^	0.68 ± 0.23 ^b^	1.11 ± 0.29 ^a^	<0.001
22:6n-3 DHA	0.38 ± 0.13 ^b^	0.45 ± 0.14 ^ab^	0.51 ± 0.14 ^a^	0.027
∑CLA	0.16 ± 0.09	0.14 ± 0.08	0.11 ± 0.03	0.112
∑n-6 PUFA	34.33 ± 2.75 ^a^	33.52 ± 1.15 ^a^	30.43 ± 1.96 ^b^	0.004
∑n-3 PUFA	3.18 ± 0.15 ^c^	5.32 ± 0.85 ^b^	9.11 ± 0.87 ^a^	<0.001

SFA = saturated fatty acids; MUFA = monounsaturated fatty acids; PUFA = polyunsaturated fatty acids; LA = linoleic acid; ALA = α-linolenic acid; AA = arachidonic acid; EPA = eicosapentaenoic acid; DPA = docosapentaenoic acid; DHA = docosahexaenoic acid; CLA = Conjugated Linoleic Acids. C = control group; L2.5% = group supplemented with 2.5% EL; L5% = group supplemented with 5% EL. Different letters on the same row mean significant differences for *p* < 0.05.

**Table 7:** Nutritional index of principal fatty acids on minced meat of rabbits fed different diets and TBARS to evaluate lipid oxidation.

	C	L2.5%	L5%	*p* Value
n-6/n-3	10.80 ± 0.98 ^a^	6.30 ± 1.62 ^b^	3.34 ± 0.75 ^c^	<0.001
SFA/PUFA	0.931 ± 0.090 ^a^	0.885 ± 0.051 ^ab^	0.820 ± 0.072 ^b^	0.008
TI index	0.891 ± 0.092 ^a^	0.764 ± 0.136 ^a^	0.576 ± 0.085 ^b^	0.005
AI index	0.510 ± 0.053 ^a^	0.497 ± 0.028 ^a^	0.451 ± 0.034 ^b^	0.002
PI index	60.80 ± 6.57 ^b^	64.37 ± 4.14 ^ab^	68.29 ± 3.72 ^a^	0.028
TBARS	0.045 ± 0.006 ^b^	0.057 ± 0.008 ^a^	0.058 ± 0.007 ^a^	0.004

SFA = saturated fatty acids; MUFA = monounsaturated fatty acids; PUFA = polyunsaturated fatty acids; AI = atherogenic index, (12:0 + 4 × 14:0 + 16:0)/(∑MUFA + ∑PUFA); TI = trombogenic index, (14:0 + 16:0 + 18:0)/ (0.5 × ∑MUFA + 0.5 × ∑n-6 PUFA + 3 × ∑n-3PUFA + n-3/n-6); PI = peroxidation index (0.025 × ∑monoenoic acids%) + (∑dienoi acids%) + (2 × ∑trienoic acids%) + (4 × ∑tetraenoic acids%) + (6 × ∑pentaenoic acids%) + (8 × ∑hexaenoic acids%). TBARS = substances reactive to thiobarbituric acids (mg di malondialdehyde/kg of meat). C = control group; L2.5% = group supplemented with 2.5% of EL; L5% = group supplemented with 5% of EL. Different letters on the same row mean significant differences for *p* < 0.05.

## Data Availability

The original contributions presented in this study are included in the article. Further inquiries can be directed to the corresponding author.

## Supplementary Materials

The following supporting information can be downloaded at: https://www.mdpi.com/article/10.3390/foods14101778/s1, Figure S1: Distribution chart of sensory analysis on the different dietary groups; Figure S2: Box plot of sensory analysis on the different dietary groups.
