# Supplementary material for: Growth Performance, Carcass Traits and Meat Quality in Rabbits Fed with Two Different Percentages of Extruded Linseed
Source: Foods. 2025 May 16;14(10):1778. doi: 10.3390/foods14101778 (PMC12111155; doi:10.3390/foods14101778)
Supplement: Supplementary file 1 [file foods-14-01778-s001.zip › foods-3586008-supplementary.pdf]

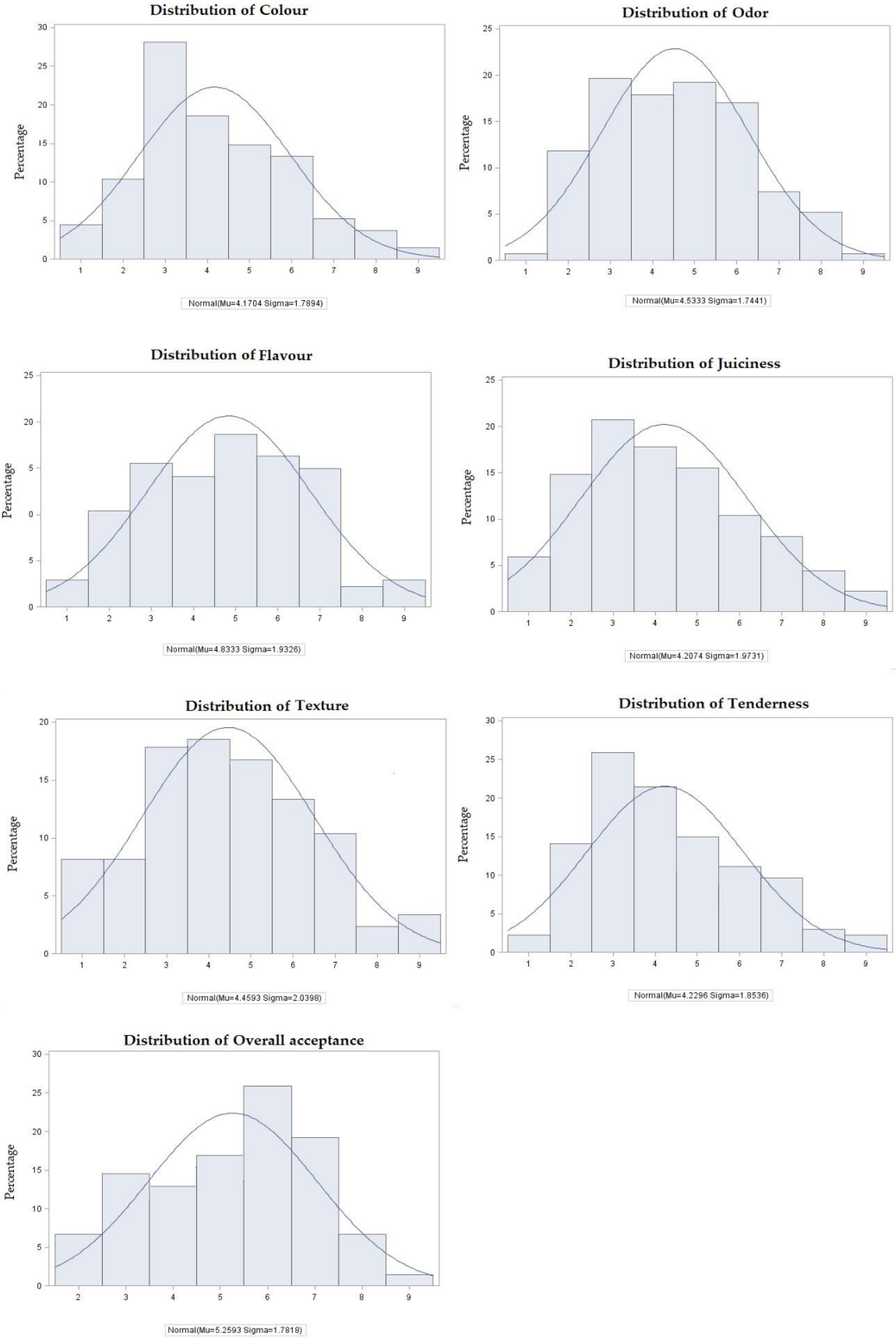

Figure S1. Distribution chart of sensory analysis on the different dietary groups.

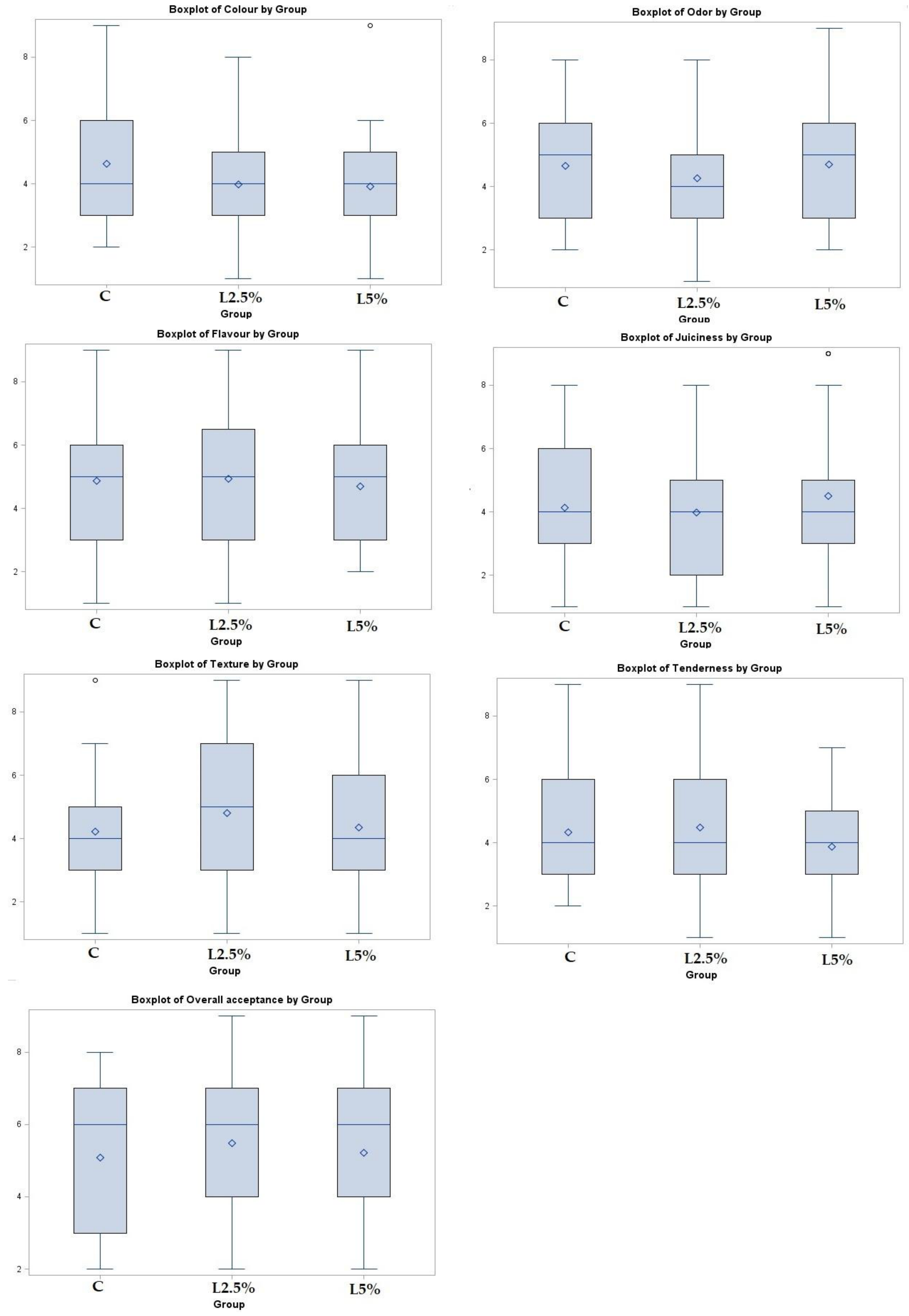

Figure S2. Box plot of the sensory analysis on the different dietary groups.
